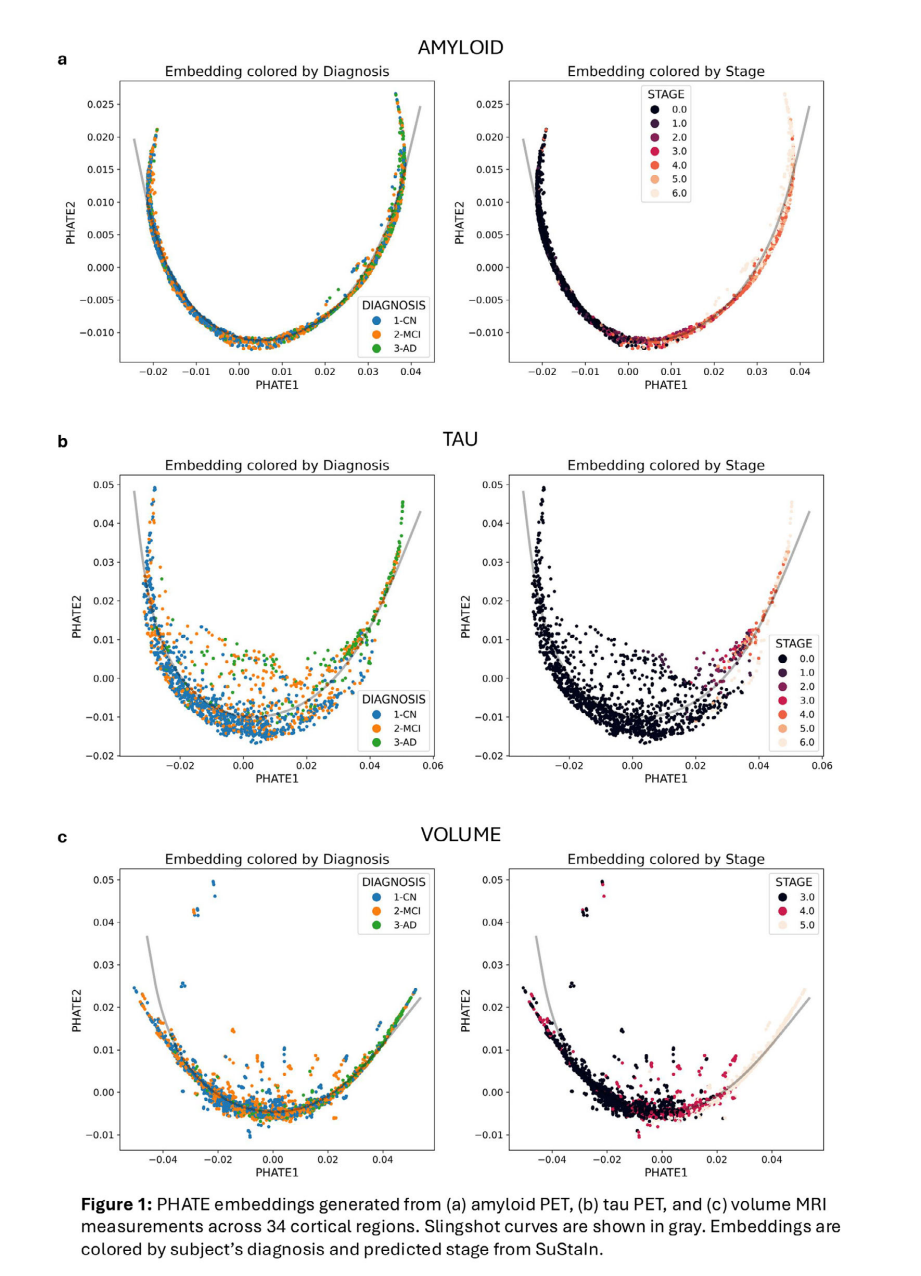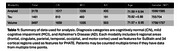# Staging and Pseudotime Inference of Alzheimer's Disease Progression

**DOI:** 10.1002/alz70856_098857

**Published:** 2025-12-24

**Authors:** Akira A Nair, Zixuan Wen, Zexuan Wang, Jingwen Yan, Andrew J. Saykin, Heng Huang, Paul M. Thompson, Christos Davatzikos, Li Shen

**Affiliations:** ^1^ University of Pennsylvania, Philadelphia, PA, USA; ^2^ Indiana university Indianapolis, Indianapolis, IN, USA; ^3^ Indiana University, Indianapolis, IN, USA; ^4^ University of Maryland, College Park, MD, USA; ^5^ University of Southern California, Los Angeles, CA, USA

## Abstract

**Background:**

Alzheimer's disease (AD) is a progressive neurodegenerative disorder marked by amyloid‐beta plaques (A), neurofibrillary tangles (T), and neuronal loss (N), commonly abbreviated A/T/N. Understanding the spatial progression of neurodegeneration is key to predicting disease trajectories and outcomes. Using Positron Emission Tomography (PET) and Magnetic Resonance Imaging (MRI) data from the Alzheimer's Disease Neuroimaging Initiative (ADNI), this study explores the staging and pseudotime of AD patients using A/T/N biomarkers.

**Method:**

We collect cortical measurements from ADNI corresponding to A/T/N modalities (Table 1). For each of the three modalities, we apply PHATE, a dimensionality reduction technique for visualizing trajectories of disease progression. We translate PHATE embeddings into 1D pseudotime values using Slingshot, an algorithm that fits principal curves to the embeddings and orthogonally projects the points onto the fitted curves. We independently run SuStaIn, a machine learning algorithm that predicts patient stages and biomarker sequences underlying disease progression. Finally, we compare SuStaIn's stage predictions with pseudotime values from PHATE and Slingshot to test disease progression robustness and generate hypotheses for biomarker events driving underlying disease progression for each imaging modality.

**Result:**

We observe that across all three modalities, the predicted SuStaIn stages are closely associated with the pseudotime values inferred by PHATE and Slingshot (Figure 1), with later stages corresponding to higher pseudotime values. Among the three modalities, amyloid PET and tau PET exhibit the strongest trajectory alignments, while MRI‐based volume shows a slightly weaker alignment. We also observe agreement between predicted biomarker event sequences from SuStaIn and PHATE pseudotime‐informed event‐based models. Pseudotimes of amyloid and tau modalities are moderately correlated (r=0.55); however, SuStaIn suggests different biomarker events driving their progressions.

**Conclusion:**

Integrative analysis of multiple computational methods allows for higher confidence in disease progression timings. Our results suggest that amyloid and tau‐associated biomarkers follow distinct trajectories in the cortex, and that different computational approaches independently arrive at similar results. While this work generates sequences of staging events and pseudotime values of disease trajectories, future work is needed to validate the putative biomarker events and connect pseudotime to real‐time disease progression.